# Agenesis of the corpus callosum

**DOI:** 10.4103/1817-1745.66662

**Published:** 2010

**Authors:** Sangram Singh, Saurabh Garge

**Affiliations:** Department of Paediatric Surgery, SAIMS, Indore, India

Sir,

The corpus callosum is a white matter structure connecting the cerebral hemispheres and is important in coordinating information and bilateral exchange of sensory stimuli. It is derived from the lamina terminalis in the portion of the neural tube cephalic to the rostral neuropore. Until the fourth month of gestation, only the most rostral part of the corpus callosum is formed; the caudal portion develops only after the fifth month.[1,2] Insults responsible for agenesis of the corpus callosum or varying degrees of hypoplasia of the corpus callosum are not identified. An early failure may lead to complete agenesis, whereas a later one will lead to hypoplasia.[3] There is a discrepancy in the reported incidence between autopsy series and those based on pneumoencephalographic studies. The incidence ranges from 0.7% to 5.3%.[4,5] Lacey[6] stated that the incidence of agenesis of the corpus callosum was only 0.0005% in an unselected random autopsy population; this seems to be lower than one would expect for a malformation which is so well known in the dysmorphology literature and community of specialists.

**Figure 1:**
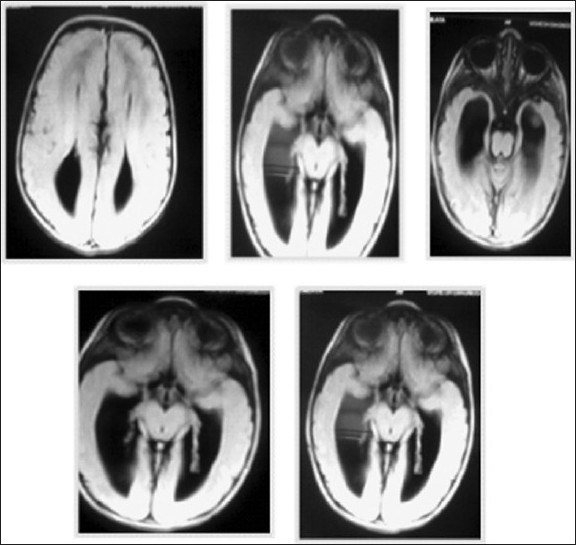
MRI showing calpocephaly and a ”rabbit ear appearance” or “devils horn appearance”

A 23-year-old primigravida was seen for ultrasonographic fetal examination, at her visit to the antenatal clinic. Antenatal USG was performed, which showed features suggestive of hydrocephalus. At 38 weeks, elective cesarean section produced a female infant weighing 2.6 kg, with Apgar scores of 7 at 1 min and 10 at 5 min. The baby looked normal at general inspection. Ultrasonic examination confirmed dilatation of the occipital horns of the lateral ventricles and again the features were told to be suggestive of mild mydrocephalus. On clinical examination, the head circumference was normal, thus suspecting some other diagnosis and she was referred for an MRI scan. There was difficulty in visualizing the corpus callosum; MRI of the head confirmed agenesis of the corpus callosum. The films showed presence of calpocephaly and rabbits horn appearance or the devils horn appearence [Figure 1]. This child illustrates the difficulty in identifying agenesis of the corpus callosum antenatally, though it was suspected by us because of the finding of an isolated dilatation of occipital horns of the lateral ventricles without any other structural anomalies being identified.

Agenesis of the corpus callosum is an uncommon cerebral malformation that has been reported in 1 in 19,000 unselected autopsies and 2.3% of children with mental retardation.[4,7] The defect may be complete or partial, depending on the stage at which callosal development is arrested.

Agenesis of the corpus callosum produces characteristic pathologic changes of the cerebral hemispheres and the ventricular system. On sagittal cuts of the MRI, the corpus callosum, cingulate gyrus, and sulcus are absent or malformed. In addition, there is a high-riding third ventricle that is open superiorly to the interhemispheric fissure. The sulci and gyri on the medial hemispheric surface have a radial or “spoke-like” configuration around the third ventricle. There is occasionally a dorsal cyst present in this space.

The lateral cerebral ventricles are displaced laterally and superiorly. In most cases, on transverse cuts of the brain, colpocephaly, or dilated occipital horns, are commonly appreciated. There is a stable, non-progressive dilatation. The reason for this enlargement is not known.[8–10] There is no evidence of obstruction along the CSF pathways. There is neither increased intraventricular pressure nor progressive ventriculomegaly. Probst bundles are longitudinal white matter tracts that indent and invaginate into the superior medial aspect of the lateral ventricles.[8–10] There are demonstrable Probst”s bundles in children with true agenesis, on a sagittal MR midline scan, which is the definitive radiological modality for evaluating agenesis of the corpus callosum. Byrd *et al*.[11] reported that MR is the best technique to evaluate the child or newborn with suspected agenesis of the corpus callosum and associated brain anomalies. He also found that ultrasound is a good screening modality of the neonatal head and can be used to demonstrate agenesis of the corpus callosum. When the findings are subtle on the ultrasound, or when agenesis of the corpus callosum is demonstrated on the ultrasonic examination, a CT or preferably an MR should be obtained to evaluate the brain for a complete outline of the corpus callosum and associated structures.

**Differential diagnosis:** Arachnoid cyst, porencephaly, hydrocephaly, and prominent septum cavum pellucidum.

The corpus callosum is phylogenetically a recent structure, and its absence is not lethal. Isolated agenesis of the corpus callosum may be either a completely asymptomatic event (found incidentally) or revealed during the course of a neurological examination by subtle deficits, such as inability to match stimuli using both hands or to discriminate differences in temperature, shape, and weight in objects placed in both hands.[12]

Persons with agenesis of the corpus callosum may have neurological problems, such as seizures (60%), intellectual impairment (70%), and psychosis.[1–3] However, these conditions are believed to be caused by abnormalities in associated cerebral anomalies rather than in the corpus callosum per se. Byrd *et al*.[11] studied a group of 105 children with a diagnosis of agenesis of the corpus callosum and reported that 26 (25%) had isolated agenesis of the corpus callosum with no associated brain anomalies. Eight of these presented with seizures which were controlled medically. Of the 105 children, 85% had symptoms and/or abnormal signs. The most common signs were macrocephaly with hydrocephalus and seizures. Postnatally, they found MR was the best radiological imaging modality for evaluating children with agenesis of the corpus callosum and associated brain anomalies. The most common associated brain anomalies (in decreasing frequency) were interhemispheric cyst with hydrocephalus, Dandy-Walker malformation, migrational disorder, absence of the inferior vermis, cephalocele, and lipoma of the interhemispheric fissure. The children who had the best prognosis without any significant neurologic sequelae were those with isolated agenesis of the corpus callosum.

The children with the worst prognosis and neurological sequelae were those with agenesis of the corpus callosum and migrational disorder with or without Dandy-Walker malformations.[11] Hence, prognosis is determined primarily by the underlying or associated malformation(s).[13]

*In utero*, the presence of hydrocephalus is usually assessed from the V/H ratio. This is the ratio of the ventricular width, as measured from the midline to the lateral wall of the lateral ventricle, to the width of the cerebral hemisphere. In agenesis of the corpus callosum, the lateral ventricles are widely separated, giving a falsely high value for the V/H ratio. The prominent posterior horns reinforce the impression of hydrocephalus as the posterior horn. V/H ratio is high and both medial and lateral borders are clearly visualized. Comstock *et al*, suggested that coronal view should be obtained if possible as this would show the characteristic concavity of the medial walls of the lateral ventricles and also possibly the absence of the corpus callosum.[14] According to Romero *et al*, upward displacement of the third ventricle is very specific.[15]

It is important to be aware of the features of agenesis of the corpus callosum. First, to distinguish it from hydrocephalus and second to instigate a search for further, more serious, anomalies with which it may be associated.
